# Murine hindlimb lymphedema model: optimization and evaluation of radiation

**DOI:** 10.1186/s13058-025-02112-8

**Published:** 2025-09-29

**Authors:** Shahnur Ahmed, Ganesh Mohan, Daniel J. Konig, Steven J. Sullivan, Christopher A. Subi-Kasozi, Angad Sidhu, Miguel Jorge, Helen C. Sinex, Marc S. Mendonca, Mithun Sinha, Aladdin H. Hassanein

**Affiliations:** 1 Division of Plastic Surgery, Department of Surgery, Indiana University School of Medicine, Indianapolis, IN US; 2 Department of Radiation Oncology, Radiation and Cancer Biology Laboratories, and Department of Medical and Molecular Genetics, Indiana University School of Medicine, Indianapolis, IN US; 3 Department of Radiation Oncology, Radiation and Cancer Biology Laboratories, Indiana University School of Medicine, Indianapolis, US

**Keywords:** Murine hindlimb, Radiation, Lymphedema, Lymphadenectomy

## Abstract

**Background:**

Post-surgical lymphedema frequently occurs following lymph node dissection. The murine tail is the most commonly used model to study secondary lymphedema. The murine hindlimb model offers a more clinically translatable approach but results in the literature have been inconsistent. The purpose of this study is to optimize the murine hindlimb lymphedema to achieve consistent results and assess the utility of radiation.

**Methods:**

C57BL/6 mice underwent either 20-Gy irradiation of one hindlimb 7 days prior to surgery (*n* = 11) or no radiation (*n* = 9). For all mice, a circumferential skin incision was created at the proximal hindlimb. Lymphatics were identified and disrupted. Popliteal lymph nodes were excised. Paw thickness was measured and near-infrared laser lymphangiography was used to assess lymphatic function.

**Results:**

The average paw thickness of the operated hindlimb in irradiated mice on postoperative day (POD) 14 was 3.5 ± 0.3 cm compared to 2.1 ± 0.05 cm on the contralateral limb (*p* = 0.0001). Lymphangiography on POD-42 showed significantly worse lymphatic function in the operated hindlimb compared to the control hindlimb (*p* = 0.003). For the non-radiated mice, the paw thickness was 2.5 ± 0.2 cm on POD-42 compared to the contralateral limb (2.1 ± 0.1 cm) (*p* = 0.0002) but smaller than radiated hindlimb group (3.2 ± 0.1 cm) (*p* = 0.0002). The nonradiated mice had significantly greater paw thickness than the control limb until POD-56 whereas the radiated mice sustained significant paw thickness to POD-90.

**Conclusion:**

Radiation of the murine hindlimb model results in sustained lymphedema compared to non-irradiated mice. The murine hindlimb lymphedema model is clinically more translatable than the murine tail model with consistent results.

**Supplementary Information:**

The online version contains supplementary material available at 10.1186/s13058-025-02112-8.

## Introduction

Lymphedema is limb swelling from lymphatic injury [1–5]. It frequently occurs following lymph node dissection and radiation during the treatment of breast cancer and melanoma [1, 2, 6]. Lymphedema affects 5 million Americans and 250 million individuals worldwide[7–10]. Although non-surgical approaches such as compression therapy and surgery (e.g., lymphovenous bypass, vascularized lymph node transfer, liposuction) can variably improve lymphedema, there is no cure[7–14]. Therefore, novel experimental approaches are important to progress care of these patients[1, 3].

The murine tail is the most commonly used model to study lymphedema and involves full thickness tail skin excision and lymphatic vessel disruption [1–3]. The murine hindlimb model offers a more clinically translatable method [6, 15–19]. However, inconsistency, including the utility of radiation, have contributed to the model being less widely adapted than the tail model [1, 6, 15–19]. The purpose of this study is to 1) optimize the murine hindlimb lymphedema model for consistency and 2) assess the effect of radiation.

## Methods

Experiments were conducted following approval by the Indiana University Institutional Animal Care and Use Committee (IACUC). Male and female C57BL/6 mice 9–10 weeks (21-23 g) were utilized (purchased from Jackson Laboratory, Bar Harbor, ME). Both male and female mice used as lymphedema affects both sexes. Paw thickness and calf thickness measurements (0.7 mm from the base of the heel) were obtained using an electronic caliper. The contralateral non-irradiated hindlimb served as control.

**Fig. 1:**
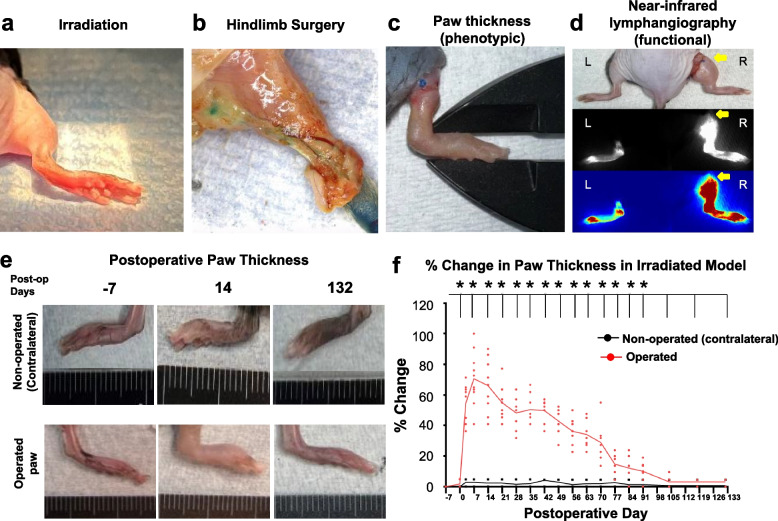
Paw thickness in irradiated murine hindlimb model The murine hindlimb model consisting of (**a**) preoperative irradiation, (**b**) circumferential skin excision, lymphatic disruption, popliteal lymphadenectomy. Assessment of postsurgical lymphedema by (**c**) paw thickness measurement and (**d**) near-infrared lymphangiography respectively. Representative images of fluorescein lymphangiography in a control (left, L) hindlimb (non-operated) and a postsurgical (right, R) hindlimb (operated). Lymphatic transection level (yellow arrow) at D42 at 12 h post-injection. Note dermal backflow. (**e**) Representative images of paw thickness on day of radiation, peak paw thickness swelling at D14, and D132 compared to the contralateral paw (*n* = 11). (**f**) Percent change in irradiated, postsurgical paw thickness measurements compared to contralateral control. *P*-value (*) < 0.05 was considered statistically significant using the Mann–Whitney U Test

Mice either underwent 20 Gy irradiation of one hindlimb (7 days prior preop, 2 mm Al filter, 2 × 2 cm field, 250 kV, 1.30 Gy/min) (Precision X-Ray Inc., North Branford, CT) or no radiation. Following inhaled isoflurane anesthesia, a circumferential skin incision was made at the proximal hindlimb (Fig. 1). Skin flaps were raised to expose hindlimb lymphatic vessels. Popliteal lymph nodes and lymphatics were identified using intradermal injection of isosulfan blue (10 µliters) in the plantar aspect of the paw. Lymphatics were transected and popliteal lymphadenectomy performed. A 3 mm circumferential wound gap was maintained for subdermal lymphatic discontinuity. Paw and calf thickness measurements were recorded on postoperative days 3, 7, and weekly.

**Fig. 2:**
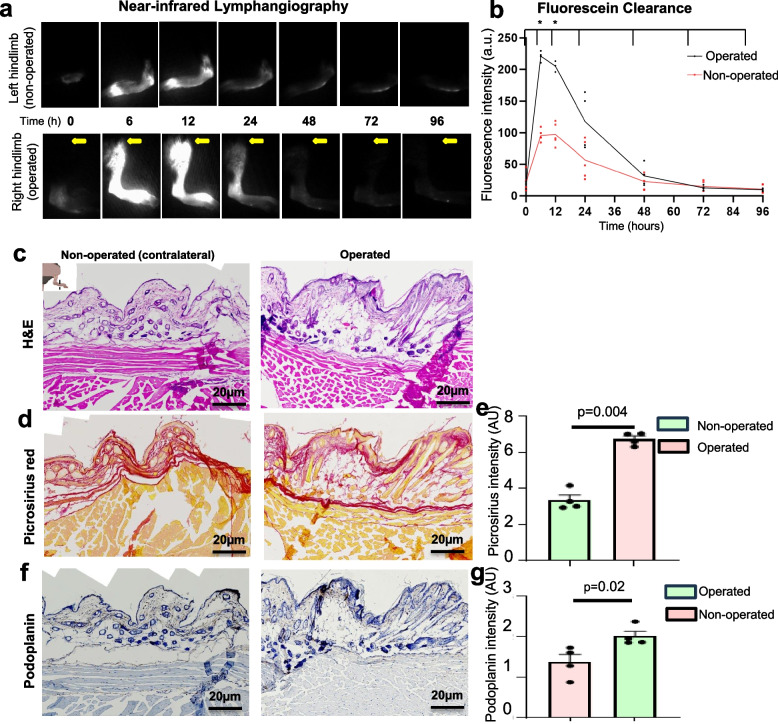
Near-infrared laser lymphangiography and histological analysis of murine hindlimb model **a-b** Fluorescein (2.5 mg/mL) was subcutaneously injected into the plantar aspect of the irradiated, lymphedematous hindlimb and non-operated hindlimb. Postinjection time lapse images were captured for 96 h. Clearance in the postsurgical, irradiated hindlimb compared to the non-operated, non-irradiated hindlimb over 96 h. *p*-value (*) < 0.05 was considered statistically significant. Representative histology images of murine paw tissue.**c** Hematoxylin and eosin (H&E) exhibits increased dermal thickness in the operated hindlimb mice. **d-e** Picrosirius red shows greater collagen and fibrosis in the radiated, operated paw compared to the contralateral nonirradiated, non-operated control. **f-g** Podoplanin exhibits greater lymphatic staining in the operated model at D35

Near-infrared laser lymphangiography was used to assess lymphatic function (Fig. 2) [1]. Fluorescein (10 µL, 2.5 mg/mL) was injected into the subdermal plantar paw. Fluorescent imaging was captured over 96H using near-infrared laser (SPY Fluorescence Imaging, Stryker, Kalamazoo, MI, USA), measured in arbitrary fluorescent units (AFU), and analyzed using ImageJ (Bethesda, MD, USA). Fluorescein clearance was determined by plotting the fluorescence intensity as a function of time. Histological examination included decalcified murine paw tissues (10% formalin fixation, paraffin embedded). Histology samples were sectioned in 3–5 μm intervals and stained with hematoxylin and eosin (H&E), podoplanin, and picrosirius red at D35. Although podoplanin, PROX1 and LYVE1 are frequently utilized as markers for lymphatic endothelial cells, podoplanin is regarded as a more specific marker for mature lymphatic vessels [20]. PROX1, though crucial for lymphatic development, is also expressed in immature lymphatic structures, while Podoplanin expression is more consistently associated with mature lymphatic endothelial cells [21]. Further, the expression of podoplanin is more limited to lymphatic endothelium in comparison to LYVE1, which may also be present on various other cell types such as certain macrophages and some tumor cells [22].

All methods were carried out in accordance with IACUC guidelines and regulations. All methods were reported in accordance with ARRIVE guidelines. Statistical analyses were performed using GraphPad Prism (GraphPad Software; Boston, MA). Parametric continuous variables were compared using paired samples *t*-tests. Non-parametric continuous variables were calculated using Mann–Whitney U tests. Two-tailed values of *p* < 0.05 were considered statistically significant.

## Results

**Fig. 3:**
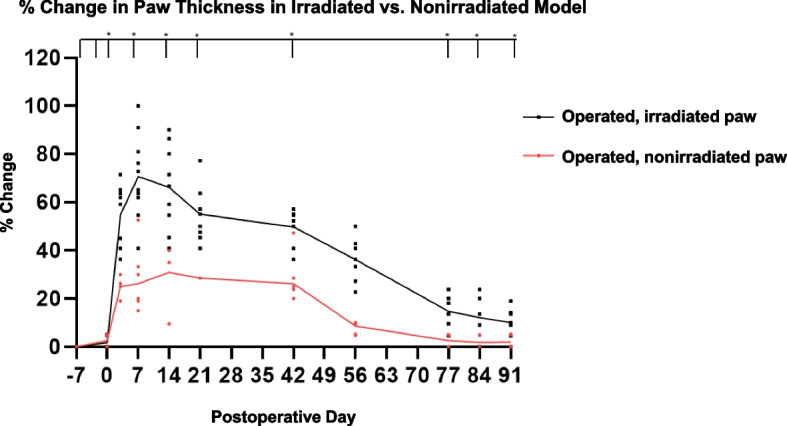
Paw thickness comparison in the irradiated and nonirradiated murine hindlimb model Percent change of paw thickness in irradiated, operated paw compared to the nonirradiated, operative paw from the day of radiation to postoperative D90

Eleven mice underwent 20-Gy irradiation of one hindlimb seven days prior to surgery and nine mice had no preoperative radiation. For the irradiated mice, the average paw thickness of the postsurgical hindlimb on D14 was 3.5 ± 0.3 cm compared to 2.1 ± 0.1 cm on the contralateral limb (CL) (*p* = 0.0001) (Fig. 3). The average calf thickness of the postsurgical hindlimb was 5.3 ± 0.6 cm on D14 compared to 4.2 ± 0.1 cm for the CL (*p* = 0.002). At D90, the average paw thickness of the irradiated, postsurgical hindlimb was 2.4 ± 0.1 cm compared to 2.1 ± 0.1 cm for the CL (*p* = 0.01). There was no difference in paw thickness of the operated hindlimb from the day of radiation to the day of surgery in irradiated mice (*p* = 0.62) and no radiation-induced necrosis. Lymphangiography at 24-h postinjection on D42 demonstrated a signal intensity of 97.7 ± 28.5 AU in the postsurgical hindlimb compared to 33.6 ± 6.2 AU in the CL (*p* = 0.003). There was no difference in dye clearance on lymphangiography at 24-h postinjection between the operated hindlimb before procedures and the control limb (*p* = 0.56) (Supplemental Figure 1). Immunohistochemistry exhibited abundance of lymphatic endothelial-specific marker podoplanin in the postsurgical hindlimb compared to the contralateral nonirradiated control at D35 (*p* = 0.02).

On D14, the average paw thickness in mice that did not undergo radiation was 2.6 ± 0.2 cm compared to 2.1 ± 0.2 cm on the CL (*p* = 0.0022) and compared to 3.5 ± 0.3 cm in mice with radiation (*p* = 0.0003). The average paw thickness in non-irradiated mice was 2.5 ± 0.2 cm on D42 was greater than the CL (2.1 ± 0.1 cm) (*p* = 0.0002) but smaller than hindlimbs that underwent radiation (3.2 ± 0.1 cm) (*p* = 0.0002). At D56, the average paw thickness in mice that did not undergo radiation was 2.2 ± 0.2 cm compared to 2.1 ± 0.1 cm in the CL (*p* = 0.32) and compared to 2.9 ± 0.1 cm in mice with radiation (*p* = 0.0003). There was no significant difference in paw thickness non-irradiated, operated hindlimb D90 compared to the CL (*p* = 0.99) (Fig. 3). The follow-up time was 132 days.

## Discussion

Secondary lymphedema is characterized by chronic limb swelling from inflammation and fibroadipose deposition from lymphatic dysfunction [1, 6]. There is no cure for secondary lymphedema [1, 6]. Clinically, patients with malignancies such as breast cancer or melanoma have high risk of developing secondary lymphedema in the limb after lymph node dissection and radiation [1]. The mouse tail is a widely accepted animal model to study lymphedema that includes full-thickness tail skin excision and lymphatic disruption [1]. While the mouse tail model has been considered a standard animal lymphedema model, it is limited by anatomical differences compared to humans that limits its clinical transability to human lymphedema [6, 15]. The mouse hindlimb model, while not as comprehensively studied, may offer superior clinical relevance as a secondary lymphedema model compared to the mouse tail model although variability exists with approach of lymphatic disruption and use of radiation [1, 6, 15–19, 23–28].

In this study, consistent results of sustained paw thickness at 90 days postoperatively were achieved using a preoperative dose of 20 Gy radiation to the proximal hindlimb one week prior to surgery, lymphatic disruption, and popliteal lymphadenectomy compared to a non-irradiated hindlimb. Average paw thickness returned to baseline level at POD-97 in the irradiated mice. The surgical approach of lymphatic disruption varies in existing literature consisting of lymphatic vessel transection and popliteal lymphadenectomy with or without subinguinal lymph node removal with follow-up time ranging between 14 to 182 days among studies that included radiation [6, 15, 17–19, 23, 26]. A study that assessed 3 groups of mice that underwent lymphatic vessel cauterization, popliteal lymphadenectomy, and up to 9 Gy of total perioperative radiation with 182-day follow-up did not find consistent paw thickness swelling using water displacement at the end of the study [16]. While our study follow-up time was 132 days, increased paw thickness using electronic caliper measurements was present for 90 days postoperatively following a single dose of 20 Gy radiation to the proximal mouse hindlimb. Other established hindlimb models have included subinguinal lymph node dissection in addition to popliteal lymphadenectomy to demonstrate a sustained hindlimb lymphedema model [15]. Our study may optimize a mouse hindlimb lymphedema model that minimizes surgical dissection required for sustained paw swelling in murine hindlimb. Rodent lower limb lymphatic drainage is typically divided into distinct lymphosomes, with the distal hindlimb draining primarily into the popliteal lymph nodes and the remaining lower limb areas draining prominently to the inguinal lymph nodes [29]. In mice, due to their smaller body size, removing the popliteal lymph nodes along with supplementary procedures usually induces sufficient edema [29]. Rats may compensate through alternative lymphatic drainage pathways including the inguinal lymph nodes when only the popliteal nodes are removed [30].

Our study offers clinically relevant optimization of the murine hindlimb lymphedema model, resulting in consistent and sustained limb swelling up to 90 days post-surgery. While previous studies have explored radiation-based models, our murine hindlimb model distinguishes itself by offering a simplified protocol that avoids complex surgical procedures or fractionated radiation dosing, thereby improving reproducibility and translational value [15, 18, 19]. Radiation of the murine hindlimb has been described as an additional component to sustain paw swelling with variability in radiation dosing and timing [6, 18, 19, 23]. Murine hindlimb irradiation ranged between 4.5 Gy to 45 Gy in previously reported studies and was delivered either 3 to 10 days preoperatively or up to 40 days postoperatively [15]. We delivered a single dose of 20 Gy seven days prior to the murine proximal hindlimb, which led to sustained paw swelling up to 90 days compared to 56 days in a non-irradiated model in our study. The effect of radiation may prolong the duration of paw swelling based on our study and has been consistent with previously reported findings in other hindlimb model protocols [6]. In addition to delivery of radiation to the hindlimb, our study shows that there is no effect on radiation on paw or calf thickness prior to surgery, which has not yet been elucidated based on previous studies assessing the murine hindlimb lymphedema model [6, 15, 16]. Previous studies that have used radiation doses lower than 20 Gy have had inconsistent results of paw swelling [16]. Radiation doses to the murine hindlimb up to 45 Gy have had radiation skin necrosis [26]. Our model includes a single dose of 20 Gy in addition to lymphatic disruption in the murine hindlimb, evaluation of radiation, and 132-day follow-up which differs from prior long-term models that use similar radiation doses and surgical methods [6, 15, 18, 19]. Selection of single dose radiation at 20 Gy may allow for sustained lymphedema without skin or soft tissue complications following radiation delivery [6, 15, 26].

The murine hindlimb lymphedema has previously been inconsistent. Studies that have included radiation and lymphatic vessel transection with popliteal lymphadenectomy have exhibited follow-up time of 14–182 days [6, 15, 17–19]. Radiation prolongs the duration of paw swelling in our study, which is consistent with other reports [6, 15]. Increased podoplanin expression has been associated with inflammation and acute injury of lymphatics [31]. Our findings show that there is no effect on radiation on paw or calf thickness prior to surgery, which has not previously been shown in this model [6, 15, 16]. Our study may optimize a mouse hindlimb lymphedema model that delivers radiation and minimizes surgical dissection required for sustained paw swelling in murine hindlimb.

## Conclusion

Radiation of the murine hindlimb model results in sustained lymphedema compared to non-irradiated mice. The murine hindlimb lymphedema model is clinically more translatable than the murine tail model and includes limb lymphatic vessel disruption, popliteal lymphadenectomy, and ideally radiation for consistent results with lymphedema sustained for 90 days.

## Supplementary Information

Supplementary Material 1: Figure 1. Fluorescein clearance was measured pre-operatively in the operated hindlimb and the non-irradiated control hindlimb over 96 hours.

## Data Availability

All relevant data are within the paper.
