# Supplementary material for: Murine hindlimb lymphedema model: optimization and evaluation of radiation
Source: Breast Cancer Res. 2025 Sep 29;27:168. doi: 10.1186/s13058-025-02112-8 (PMC12481953; doi:10.1186/s13058-025-02112-8)
Supplement: Supplementary file 1 — Supplementary Material 1: Figure 1. Fluorescein clearance was measured pre-operatively in the operated hindlimb and the non-irradiated control hindlimb over 96 hours. [file 13058_2025_2112_MOESM1_ESM.pptx]

## Slide 1
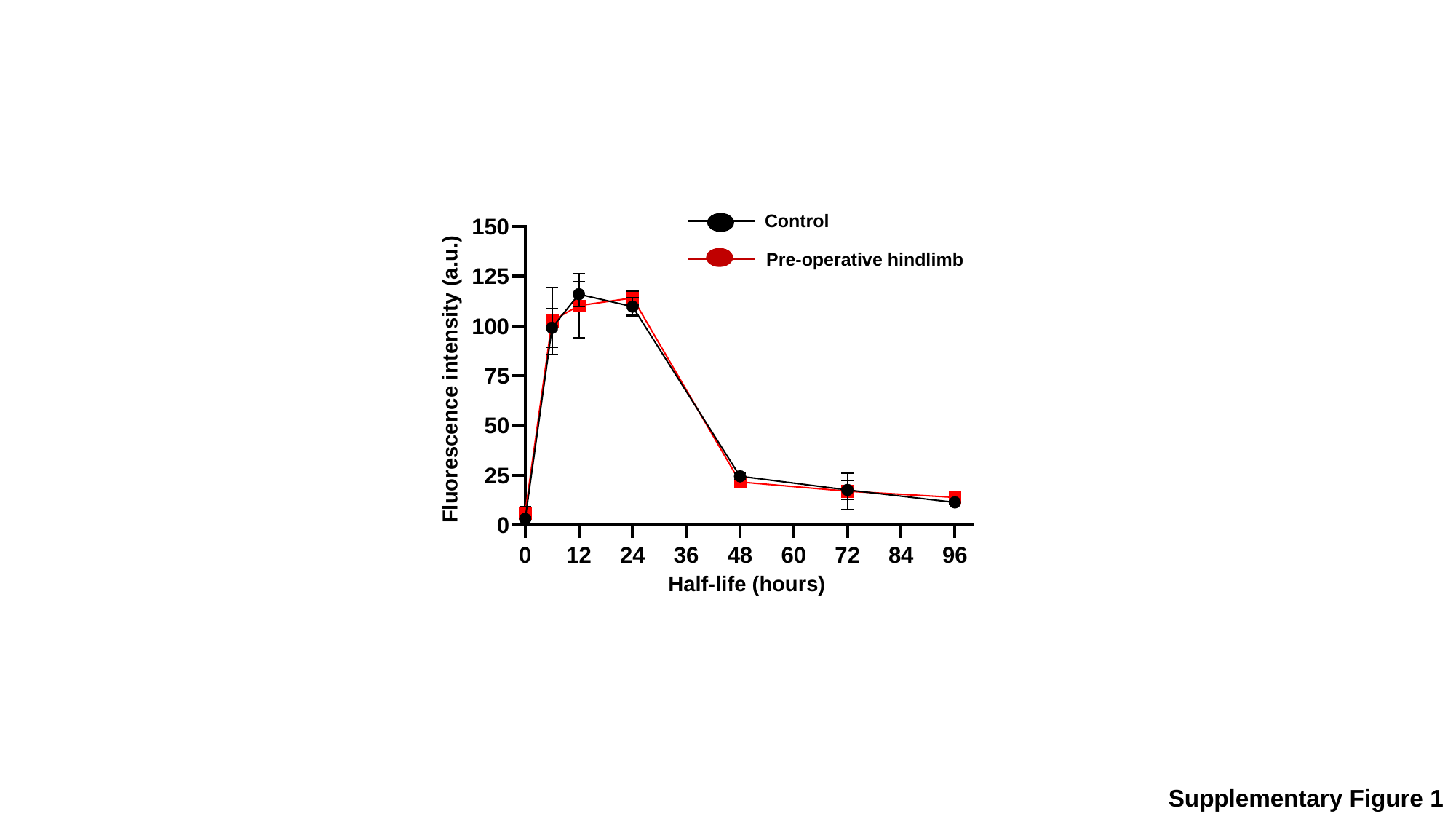

Control
Pre-operative hindlimb
Fluorescence intensity (a.u.)
Half-life (hours)
Supplementary Figure 1
12
0
6
24
48
72
96

## Slide 2
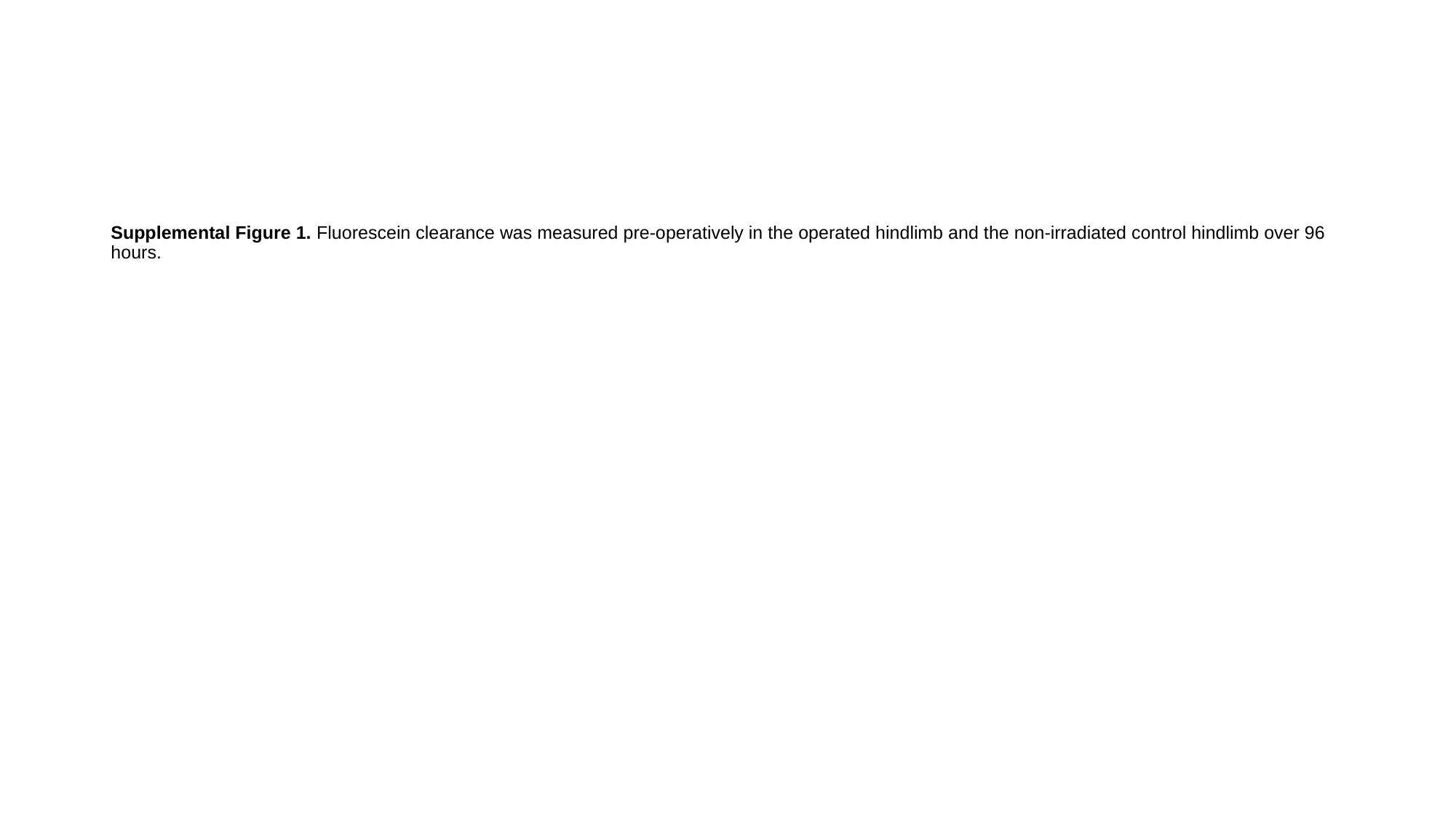

Supplemental Figure 1. Fluorescein clearance was measured pre-operatively in the operated hindlimb and the non-irradiated control hindlimb over 96 hours.
